# Risk of psoriasis in people with hidradenitis suppurativa: A systematic review and meta-analysis

**DOI:** 10.3389/fimmu.2022.1033844

**Published:** 2022-12-01

**Authors:** Shuo-Yan Gau, Ivan Arni C. Preclaro, James Cheng-Chung Wei, Chien-Ying Lee, Yu-Hsiang Kuan, Yu-Ping Hsiao, Sin-Ei Juang, Kevin Sheng-Kai Ma

**Affiliations:** ^1^ School of Medicine, Chung Shan Medical University, Taichung, Taiwan; ^2^ Department of Medical Education, Chung Shan Medical University Hospital, Taichung, Taiwan; ^3^ Department of Dermatology, Chang Gung Memorial Hospital, Taoyuan, Taiwan; ^4^ Drug Hypersensitivity Clinical and Research Center, Chang Gung Memorial Hospital, Taoyuan, Taiwan; ^5^ Division of Allergy, Immunology and Rheumatology, Department of Internal Medicine, Chung Shan Medical University Hospital, Taichung, Taiwan; ^6^ Graduate Institute of Integrated Medicine, China Medical University, Taichung, Taiwan; ^7^ Institute of Medicine, Chung Shan Medical University, Taichung, Taiwan; ^8^ Department of Pharmacology, Chung Shan Medical University, Taichung, Taiwan; ^9^ Department of Pharmacy, Chung Shan Medical University Hospital, Taichung, Taiwan; ^10^ Department of Dermatology, Chung Shan Medical University Hospital, Taichung, Taiwan; ^11^ Department of Anesthesiology, Kaohsiung Chang Gung Memorial Hospital and Chang Gung University College of Medicine, Kaohsiung, Taiwan; ^12^ Department of Dermatology, Massachusetts General Hospital, Boston, MA, United States; ^13^ Department of Epidemiology, Harvard T. H. Chan School of Public Health, Boston, MA, United States; ^14^ Center for Global Health, Perelman School of Medicine, University of Pennsylvania, Philadelphia, PA, United States

**Keywords:** hidradenitis suppurativa, psoriasis, meta-analysis, epidemiology, immunology

## Abstract

**Background:**

Hidradenitis suppurativa were associated with comorbidities in various organ systems. Inflammatory dermatological diseases such as pyoderma gangrenosum were reported to be associated with hidradenitis suppurativa. Nevertheless, as for the association between hidradenitis suppurativa and psoriasis, evidences were insufficient. In many studies, the association between psoriasis and hidradenitis suppurativa has been reported. However, some evidence seems to be controversial. The purpose of the systematic review and meta-analysis was to assess whether there was significant association between HS and psoriasis.

**Methods:**

On June 01, 2022, we appraised 2,795 articles from databases including PubMed, Web of Science and Embase. Search syntaxes were based on ‘hidradenitis suppurativa’ or ‘acne inversa’ with “psoriasis”, “comorbidities” or ‘epidemiology’. Synonyms were determined based on MeSH terms and Emtree. Observational results that evaluated the odds ratio for people with hidradenitis suppurativa who had psoriasis were extracted for qualitative synthesis.

**Results:**

After the selection process of the initial 2,795 studies, ten observational studies, including 3 cohort studies, 1 case-control study, and 6 cross-sectional studies, were extracted for critical appraisal. Based on the integration of 7 studies (with more than 560,000 participants included), people with hidradenitis suppurativa had a higher risk of having psoriasis, with a 2.67-fold risk (95% CI, 1.84, 3.87). The association remained in the sensitivity analyses utilizing strict adjustment models. In the analysis that only included studies with a similar study design and adjustments in obesity-related factors, the risk of people with hidradenitis suppurativa having psoriasis was 3.24 (95% CI, 2.27, 4.62). In male patients with HS, the risk of having psoriasis was 4.30-fold higher than male patients without HS (95% CI, 2.37, 7.78). Likewise, in an analysis including 3 cross-sectional studies, the risk of female HS patients having psoriasis was 3.94-fold higher than female HS-free patients (95% CI, 2.34, 6.63).

**Conclusions:**

The co-occurrence of hidradenitis suppurativa and psoriasis can greatly increase the burden of the disease. Psoriasis could be one of the critical comorbidities of hidradenitis suppurativa and should be recommended for future screening and follow up. The association between the two diseases should be kept in mind in managing hidradenitis suppurativa patients. More prospective studies are needed to establish the true magnitude of the association between psoriasis and hidradenitis suppurativa.

## Highlights

Identifying comorbidities in hidradenitis suppurativa (HS) is critical to give patients recommendations for the screening for comorbidities. Recently, HS has been reported to have a strong association with psoriasis. However, some evidence seemed to be controversial and the current guidelines for comorbidity screening recommendation have insufficient information regarding the HS-psoriasis association. In the current systematic review and meta-analysis, we report that, based on the integration of 7 studies with more than 560,000 participants included, people with HS had a more than 2.6-fold risk of having psoriasis. The significance of the association remained in the sensitivity analyses using a strict adjustment model and different study design. In the analysis that only included studies with similar study design and adjusted for obesity-related factors, the risk of people with HS having psoriasis was 3.24 (95% CI, 2.27, 4.62). The coexistence of HS and psoriasis can greatly increase the burden of the disease that may require aggressive treatment. Further prospective studies are needed to establish the magnitude of the association between psoriasis and HS.

## Introduction

Hidradenitis suppurativa (HS) is a chronic, inflammatory skin disorder that affects apocrine units. It is characterized by nodules and cysts that form sinus tracts, which is seen prominently in intertriginous areas (1). Psoriasis is a chronic inflammatory disorder that prominently affects skin and joints. Erythematous papules and plaques topped with thick micaceous scales are typical and are seen more commonly on the extensor surfaces of the extremities (2). Despite their differences in skin manifestations, both chronic disorders share common risk factors, such as obesity and cigarette smoking (3), heavy burden on quality of life (4, 5) and common inflammatory mediators, such as the interleukin (IL) 12-IL 23 pathway, IL-17 interactions, and tumor necrosis factor (TNF) alpha (1, 6, 7).

Several reports documented associations with different autoimmune diseases and lifestyle diseases in both HS and psoriasis. Diseases including metabolic syndrome, diabetes mellitus, inflammatory bowel disease, and spondyloarthropathies, were reported to be associated with both HS and psoriasis (8, 9). Even though sharing similar comorbidities, the association between HS and psoriasis was not yet confirmed. Recently, there were studies reporting potential association between psoriasis and HS (10). However, evidences from different studies appeared to be controversial (11, 12). Despite the evidence on the association between the two diseases, studies were still limited to determine its magnitude of association. Furthermore, in the current guidelines for screening for HS comorbidities, the evidences regarding HS-psoriasis association remained insufficient (13). The purpose of the study is to assess whether there is significant association between HS and psoriasis. The authors performed a systematic review and meta-analysis of cases compared to controls to determine the association between HS and psoriasis.

## Methods

### Literature searching, process of screening and eligibility criteria for studies

In the current meta-analysis, the Preferred Reporting Items for Systematic Reviews and Meta-analyses (PRISMA) was utilized to ensure the screening process being precise and objective (14). Real-world observational studies that evaluated the odds ratio of psoriasis in people with HS, including studies in cohort, case-control and cross-sectional study design, were targeted for data extraction in systematic review and meta-analysis.

On June 01, 2022, we appraised 2,795 articles from databases including PubMed, Web of Science and Embase. Search syntaxes were based on ‘hidradenitis suppurativa’ or ‘acne inversa’ with “psoriasis”, “comorbidities” or ‘epidemiology’. Synonyms were determined based on MeSH terms and Emtree. The detailed study protocol on the search strategy and inclusion criteria were provided in the **Supplementary Information.**

In this study, our aim was to provide integrated real-world evidence based on observational results. The following are the exclusion criteria: (1) studies that had no relationship with HS, (2) studies related to HS but not focusing on HS related comorbidities related to HS, (3) studies focused on genetic roles or pathophysiologic mechanisms in HS, and (4) studies reporting coexistence but not containing proper comparative groups. In the screening process, the study language and ethnicity of the participants were not established as specific exclusion criteria. Conference abstracts were also included in the selection process to address potential publication bias.

### Data extraction and assessment of risk of bias

Study characteristics were reported in **Table 1**. Baseline information from the extracted studies, including the definition of HS diagnosis, the number of participants, the gender ratio, the mean age of overall participants, was recorded. Regarding the evaluation of the risk of bias and study quality, the Newcastle-Ottawa scale was used (22).

**Table 1:** Baseline characteristic of included observational studies.

Author	Study design	Year	Location	Definition of HS patients	Definition of outcome event	Female percentage case/control (%)	Mean age (yo) case/control	Adjustment	No. of participants	NOS
Ingram et al. (11)	case-control (proxy cases)	2018	UK	CPRD Read code algorithms identifying clinical features. Validated by subsequent questionnaire	Read codes in CPRD	NA	NA	age, sex and registration at the same primary care practice	163711	7
Ingram et al. (11)	case-control (physician-diagnosed cases)	2018	UK	Read codes in CPRD and ICD-10-based diagnosis	Read codes in CPRD	NA	NA	age, sex and registration at the same primary care practice	117896	7
Lee et al. (12)	cross sectional	2018	Korea	at least two documented physician contacts based on ICD-10 diagnosis	at least 2 physician validated ICD-10-based diagnosis	38.7/38.7	33.6/33.6	age, sex, socioeconomic status and the presence of diabetes, hypertension and dyslipidemia.	171096	6
Kimball et al. (15)	cohort (moderate HS)	2018	US	at least two ICD-9-based diagnoses	medical claims recorded	74/74	42.19/42.19	age, sex, region of residence, and healthcare plan	4584	7
Kimball et al. (15)	cohort (severe HS)	2018	US	at least two ICD-9/ICD-10-based diagnosis diagnoses and experienced at least one of the disease severity indicators	medical claims recorded	71/71	42.19/42.19	age, sex, region of residence, and healthcare plan	6130	7
Andersen et al. (10)	cross sectional	2020	Denmark	ICD-10-based diagnosis HS	ICD-10-based diagnosis	87.5/NA	NA	NA	440 HS group/NA for control	5
Schneeweiss et al. (16)	cohort	2020	US	at least three ICD-9/ICD-10-based diagnosis or more than one HS diagnosis by a dermatologist	NA	75.5/76.7	36.6/37.6	age, sex, region, number of outpatient visits, number of unique medications, use of systemic biologic or nonbiologic immunomodulatory agents, comorbidities, combined comorbidity score	121744	6
Schneeweiss et al. (17)	cohort	2021	US	at least three ICD-9/ICD-10-based diagnosis on or more than one HS diagnosis by a dermatologist	NA	75.5/76.6	36.48/37.93	age, sex, health care utilization patterns, comorbidities, comedications, Gagne combined comorbidity score	132896	6
Hua et al. (18)	cross sectional	2021	US	at least two ICD-9/ICD-10-based diagnosis	ICD9/10 diagnosis record	73.6/73.6	40.7/40.7	age, sex, race, index date, obesity, tobacco use	150337	7
Kirsten et al. (19)	cross sectional	2021	German	Assessed by dermatologist based on clinical exams and findings	Assessed by dermatologists	38.6/47.8	(pooled)43.6	NA	20112	5
Prens et al. (20)	cross sectional	2021	Netherlands	Self-reported by validated questionnaire	Self-reported by validated questionnaire	73.5/60.1	52.1/56.0	age, sex, BMI, smoking status and socioeconomic status	56084	7
Sokumbi et al. (21)	cross sectional	2022	US	ICD-10 diagnosis and validation of dermatologists	ICD-10 diagnosis	73.0/73.0	35.4/35.5	Age, sex, index date	2320	6

HS, Hidradenitis suppurativa; ICD-9, International Classification of Diseases, 9th Revision; ICD-10, International Classification of Diseases, 10th Revision;

y/o, years old; NA, not available; NOS, Newcastle-Ottawa Scale.

### Statistical analysis

In the current study, Review Manager 5.4 (Cochrane, London, UK) was used to perform statistical analyses. The results of the observational studies evaluating HS-psoriasis relationship were extracted and analyzed to present the pooled odds ratio of the association. Given that the clinical heterogeneity could possibly exist, we utilized the random-effects model in all of the qualitative synthesis. The odds ratio (OR) was evaluated based on 95% confidence interval (95% CI). For the pooled studies in each analysis, heterogeneity was determined by applying the value of I (2). If the presented I (2) value was greater than 75%, there could be high heterogeneity within pooled studies (23).

## Results

### Baseline characteristics and the quality evaluation of included observational studies

**Figure 1:**
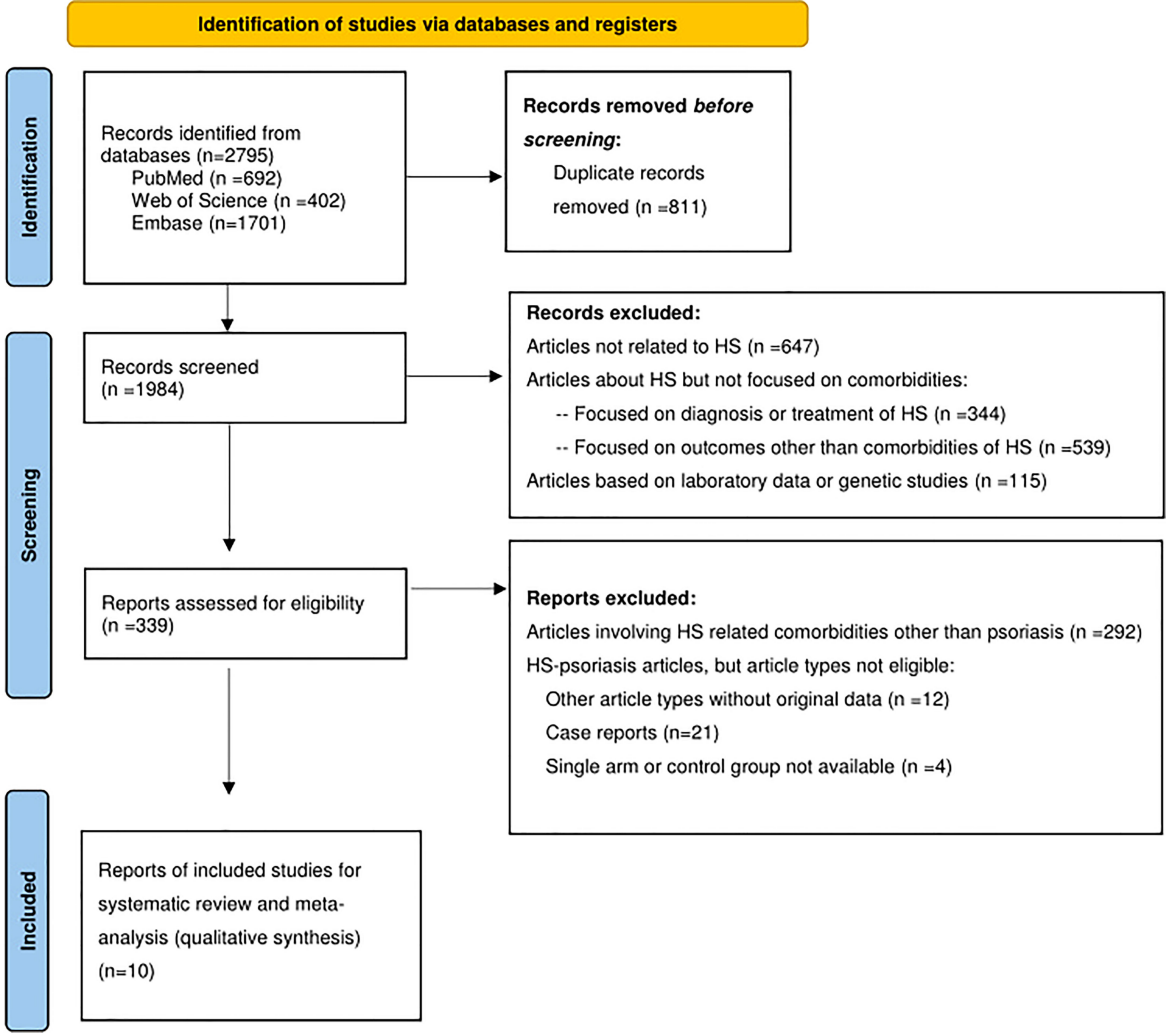
PRISMA study flowchart.

The PRISMA flow diagram was reported in **Figure 1**. Initially, there were 2,795 studies identified from the databases. After the screening process excluding ineligible studies, we included 10 observational studies for critical appraisal. This includes 3 cohort studies (15–17), 1 case-control study (11) and 6 cross-sectional studies (10, 12, 18–21). Within the included studies, only one of them were originated from Asia, whereas most of the studies were originated from Europe (4out of 10 studies) and the United States (5 out of 10 studies). In most of the studies, the definition of HS was based on ICD-9/10 diagnostic codes or based on the evaluation of a dermatologist. Given that some of the studies did not provide sufficient information on the criteria for psoriasis diagnosis, detection bias may be present in the included studies.

### Qualitative synthesis: Odds ratio of HS-psoriasis

**Figure 2:**
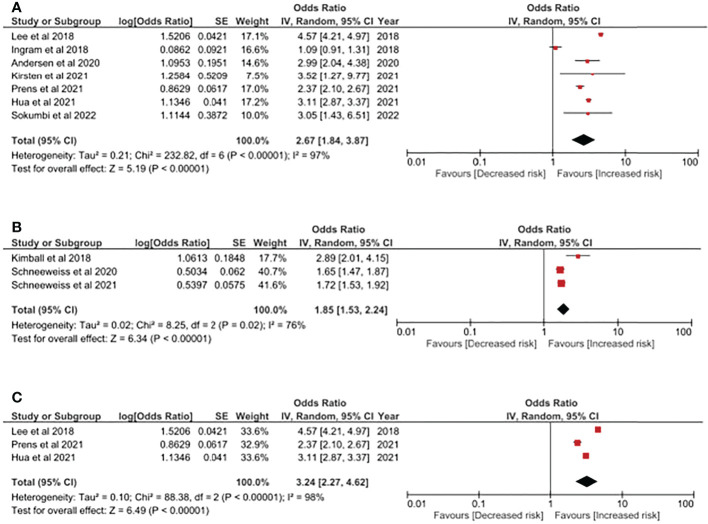
**(A)** Odds ratio of psoriasis in people with Hidradenitis Suppurativa. **(B)** Sensitivity analysis: Odds ratio of psoriasis in people with Hidradenitis Suppurativa (evidences based on cohort study design). **(C)** Sensitivity analysis: Odds ratio of psoriasis in people with Hidradenitis Suppurativa in obesity-adjusted models. Legends: Obesity-related factors could serve as critical confounding factors in the pathogenesis in both HS and psoriasis. Thereby, in this model, only studies performing adjustment in obesity-related factors (i.e: BMI, hyperlipidemia, etc.) were included.

In **Figure 2A**, the odds ratio of having psoriasis in people with HS was reported. Based on the integration of 7 studies with more than 560,000 participants included, people with HS had a higher risk of having psoriasis, with a 2.67-fold risk (95% CI, 1.84, 3.87). The heterogeneity should not be neglected since the I (2) value in the current analysis was 97%. Populations included in this analysis included patients from Asia, Europe and the United States. We have performed additional sensitivity analyses including other study design (using cohort studies only) to evaluate the association of HS and psoriasis. As reported in **Figure 2B**, the HS-psoriasis association remained after including cohort studies, with a 1.85-fold risk (95% CI, 1.53, 2.24). The I (2) value in the analysis was reported to be 76%, which could be moderate to massive heterogeneity between included studies. Furthermore, given that obesity-related factors could serve as a critical confounder in the pathogenesis of HS and psoriasis, we also conducted an additional analysis to address this bias. Therefore, in the analysis performed in **Figure 2C**, only studies with similar study design and adjustment in obesity-related factors were included. In this analysis, the risk of HS patients having psoriasis was also statistically significant, with a 3.24-fold risk (95% CI, 2.27, 4.62).

### Qualitative synthesis: Stratification analysis based on gender

**Figure 3:**
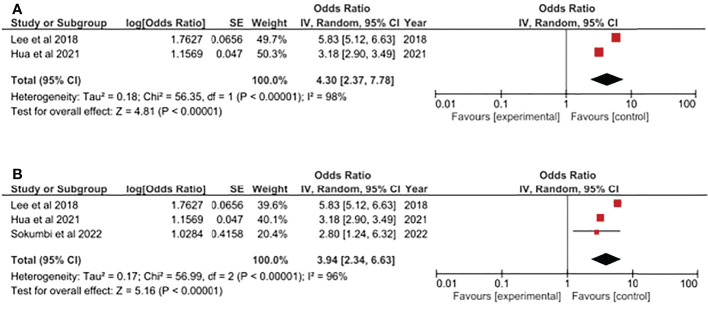
**(A)** Odds ratio of psoriasis in male population with Hidradenitis Suppurativa. **(B)** Odds ratio of psoriasis in female population with Hidradenitis Suppurativa.

Additionally, based on the current evidence, we have evaluated the HS-psoriasis association based on different genders. For HS patients, both genders presented approximately 4-fold risk of having psoriasis. In male patients with HS, the risk of having psoriasis was 4.30-fold higher (95% CI, 2.37, 7.78) (**Figure 3A**). In an analysis including 3 cross-sectional studies, the risk of female HS patients having psoriasis was 3.94-fold higher (95% CI, 2.34, 6.63) (**Figure 3B**). In both analyses, the results should be cautiously interpreted since both analyses were based on the integrated analysis of small amount of studies. Moreover, the heterogeneity might be great within included studies due to high I (2) values.

## Discussion

HS is a chronic, inflammatory disorder of the apocrine units affecting the intertriginous areas. It occurs in 6 out of 100,000 people with the highest incidence among young women aged 20 to 29 years old. Obesity and smoking history are associated with the occurrence of HS, while smoking and female sex are significantly correlated with more severe disease (24). Several disease correlations have been found including autoimmune diseases such as type 1 diabetes mellitus, rheumatoid arthritis, ankylosing spondylitis and ulcerative colitis; renal diseases; dermatological diseases such as vitiligo and alopecia areata; and metabolic diseases such as thyroid diseases, hypertension and hyperlipidemia (25–27). The present study demonstrated that patients with HS have a 2-3 times the risk of being affected with psoriasis. Among the extracted articles, the strongest correlation was observed in a study of 13,667 HS patients where there is three times the risk of developing psoriasis compared to controls (18). Moreover, in the stratification of gender, both male and female patients with HS presented the trend of higher psoriasis risk. The integrated evidence provided in the current study could possibly serve as useful reference for clinicians for giving recommendations of comorbidity screening in people with HS.

Prevalence and incidence of psoriasis were found to be high in previous studies. One Asian study reported a higher prevalence of psoriasis in patients with HS (prevalence rate=38.6) than those without HS (prevalence rate=8.9) (12). In Western populations, the trend was also observed. According to a American single-center study recruiting 1,160 HS patients and the same amount of controls, odds ratio for HS patients having psoriasis was significantly higher than controls, with an observed more than 3-fold higher risk (21). Another study has identified that the incidence of psoriasis in HS patients was approximately 6% with a greater psychological burden compared to healthy controls and without disease co-occurrence (28). In a study by Pinter et al. (29), there was a slight male predominance (1.15:1) with the first symptoms of psoriasis occurring earlier than symptoms of HS. However, for those with HS disease as its first symptoms, the severity of the disease is greater than those patients who started with symptoms of psoriasis. Then, the onset of the second disease occurs around 14.3 years after the first disease. Furthermore, the most frequent comorbidity in the cohort was obesity, psychiatric complaints and psoriatic arthritis.

In many diseases, the inflammatory state could play potential roles in the association with autoimmune or endocrinological comorbidities (30–32). The association between HS and psoriasis may be related to their common inflammatory pathways. For a long time, psoriasis was believed to be initiated by the release of TNF alpha, interferon (IFN)-alpha and IFN-beta from plasmacytoid dendritic cells (DCs). These soluble factors further enhance inflammation by activation of inflammatory DCs that secrete more TNF alpha and IL-23. The key cytokine, IL-23, is the main mediator in activating and maintaining the inflammatory cascade brought about by T helper (Th) 17 and Th22 cells (33). Parallel with this, recent studies have shown that there was also an overexpression of IL-23 on lesional and perilesional skin, and serum levels of patients with HS (34). This was further studied by Navrazhina et al. (6) in which detailed biomarkers were investigated in both HS and psoriasis. It was found that there is a significant increase in IL-17A and HGF in HS comparatively similar to psoriasis. HS was thought to be highly involved in the pathway of Th17 mechanism. As for the concentration of IL17 in blood, previous studies also reported that the mean level for HS patients was 3.68 ± 2.08 pg/mL, which was significantly higher when comparing with healthy controls, which presented mean level of serum IL17 of 2.5 ± 1.11 pg/mL (35). Moreover, in different area of HS patients, including lesioned or normal cutaneous areas, Th17-related cytokines such as IL17 and IL23 were observed to be elevated (34, 36). The similarity in the cytokines involved may explain the usefulness of biologic therapies in HS. Among biologic therapies, TNF alpha inhibitors such as adalimumab and IL-17 inhibitors such as brodalumab were found to have a positive effect in patients with HS and psoriasis (29, 37, 38).

The present study has limitations that need to be addressed. First, the meta-analysis included a small number of studies. However, the studies included have generally observed the positive association between HS and psoriasis. Second, the included literature was retrospective observational studies that may not determine the true association between HS and psoriasis. Future prospective studies are recommended to establish the association between the two. Furthermore, there were some studies at high risk of bias in outcome assessment due to the application of administrative codes for disease definition. Third, confounders could cause potential bias. In observational studies, some residual confounders might not be adjusted as covariates (39–41). Therefore, although we have tried our best to perform sensitivity analyses in integrating evidence with critical adjustment models, confounders could still exist in observational studies and the meta-analysis based on these studies. Fourth, given that most of the retrieved studies utilized administrative codes to identify psoriasis, in most of the retrieved studies for data extraction, subcategories of psoriasis were not available. In this case, we might not be able to identify which specific psoriasis phenotype is associated with higher co-occurrence of HS. However, this was described in another study where the psoriasis phenotype observed with co-occurrence of HS was guttate psoriasis and palmoplantar pustulosis (10) Future studies with larger scale were warranted to perform detailed classification of psoriasis while determining their association with HS. Fifth, among the studies retrieved, the burden of disease in patients with psoriasis and HS have not been discussed. It’s difficult to evaluate the high prevalence and odds ratio of having psoriasis could influence the patients’ quality of life. Sixth, the information of the disease activity of psoriasis was limited in the in most extracted studies of the current meta-analysis. Mean age of enrolled HS patients might be able to represent the disease activity of psoriasis. In this case, we were not able to exactly know the psoriasis onset time of patients. it’s difficult to determine whether or not the psoriasis status was early or late onset types. Finally, publication bias may be probable. Therefore, the results should be interpreted with caution.

In conclusion, the meta-analysis confirmed the strong association between HS and psoriasis. This supports the advantageous use of biologic therapies with or without HS patients due to its common inflammatory pathway. The co-occurrence of HS and psoriasis can greatly increase the burden of the disease that may require aggressive treatment. Further prospective studies are needed to establish the true magnitude of the association between psoriasis and HS.

## Data availability statement

The original contributions presented in the study are included in the article and **Supplementary Material**. Further inquiries can be directed to the corresponding authors.

## Author contributions

All the authors involved in drafting or revising the article and approved of the submitted version. Study conception and design: S-YG, Y-PH, KM, C-YL, Y-HK, S-EJ, and JW. Data analysis and demonstration: S-YG. Original draft preparation: S-YG and IP. All authors contributed to the article and approved the submitted version.

## Conflict of interest

The authors declare that the research was conducted in the absence of any commercial or financial relationships that could be construed as a potential conflict of interest.

## Publisher’s note

All claims expressed in this article are solely those of the authors and do not necessarily represent those of their affiliated organizations, or those of the publisher, the editors and the reviewers. Any product that may be evaluated in this article, or claim that may be made by its manufacturer, is not guaranteed or endorsed by the publisher.
