# Supplementary material for: Risk of psoriasis in people with hidradenitis suppurativa: A systematic review and meta-analysis
Source: Front Immunol. 2022 Dec 1;13:1033844. doi: 10.3389/fimmu.2022.1033844 (PMC9752046; doi:10.3389/fimmu.2022.1033844)
Supplement: Supplementary file 1 [file DataSheet_1.docx]

**Supplementary Information**:

1. **PICO and inclusion/exclusion criteria:**

**Participants:**

All patient with hidradenitis suppurativa (age, sex and other baseline characteristics were not limited)

**Intervention:** NA

**Comparison:**

All people without hidradenitis suppurativa (age, sex and other baseline characteristics were not limited)

**Outcomes:**

Psoriasis

**Inclusion and exclusion criteria**:

Only studies meeting the PICO will be included. Articles not evaluating the prevalence of psoriasis in hidradenitis suppurativa and those without appropriate control group will be excluded from data extraction.

1. **Searching syntaxes: Retrieved on 2022.06.01**

**Pubmed, n=692**

(hidradenitis suppurativa OR acne inversa) AND (psoriasis OR Psoriases OR Pustulosis of Palms and Soles OR Pustulosis Palmaris et Plantaris OR Palmoplantaris Pustulosis OR Pustular Psoriasis of Palms and Soles OR comorbidity OR multimorbidity OR epidemiology) NOT review

**Web of Science, n=402**

#1 (ALL=(hidradenitis suppurativa)) OR ALL=(acne inversa)

#2

((ALL=(psoriasis )) OR ALL=(comorbidity )) OR ALL=(epidemiology)

#3 (#1 AND #2 )

#4 (#3) NOT ALL=(review)

**Embase, n=1701**

#1

'hidradenitis suppurativa'/exp OR 'hidradenitis suppurativa' OR 'suppurativa' OR 'hidradenitis'/exp OR 'hidradenitis' OR 'suppurativa hidradenitis'/exp OR 'suppurativa hidradenitis'

#2

'psoriasis'/exp OR psoriasis OR 'psoriasiform dermatitis'/exp OR 'psoriasiform dermatitis' OR (psoriasiform AND ('dermatitis'/exp OR dermatitis)) OR 'psoriasiform dermatosis'/exp OR 'psoriasiform dermatosis' OR (psoriasiform AND ('dermatosis'/exp OR dermatosis)) OR 'psoriasiform lesion'/exp OR 'psoriasiform lesion' OR (psoriasiform AND ('lesion'/exp OR lesion)) OR 'psoriasiform rash'/exp OR 'psoriasiform rash' OR (psoriasiform AND ('rash'/exp OR rash)) OR 'psoriasiform skin rash'/exp OR 'psoriasiform skin rash' OR (psoriasiform AND ('skin'/exp OR skin) AND ('rash'/exp OR rash)) OR 'psoriatic epidermis'/exp OR 'psoriatic epidermis' OR (psoriatic AND ('epidermis'/exp OR epidermis)) OR 'psoriatic skin'/exp OR 'psoriatic skin' OR (psoriatic AND ('skin'/exp OR skin)) OR 'skin rash, psoriasiform'/exp OR 'skin rash, psoriasiform' OR (('skin'/exp OR skin) AND ('rash,'/exp OR rash,) AND psoriasiform) OR 'willan lepra'/exp OR 'willan lepra' OR (willan AND ('lepra'/exp OR lepra))

#3

'comorbidity' OR 'comorbidity'/exp OR comorbidity OR 'co morbidity'/exp OR 'co morbidity' OR 'multiple chronic conditions'/exp OR 'multiple chronic conditions' OR (multiple AND chronic AND conditions) OR 'concurrent chronic conditions'/exp OR 'concurrent chronic conditions' OR (concurrent AND chronic AND conditions) OR 'concurrent chronic diseases'/exp OR 'concurrent chronic diseases' OR (concurrent AND chronic AND ('diseases' OR 'diseases'/exp OR diseases)) OR 'concurrent chronic disorders'/exp OR 'concurrent chronic disorders' OR (concurrent AND chronic AND ('disorders' OR 'disorders'/exp OR disorders)) OR 'concurrent chronic health conditions'/exp OR 'concurrent chronic health conditions' OR (concurrent AND chronic AND ('health' OR 'health'/exp OR health) AND conditions) OR 'concurrent chronic illnesses'/exp OR 'concurrent chronic illnesses' OR (concurrent AND chronic AND illnesses) OR 'concurrent chronic medical conditions'/exp OR 'concurrent chronic medical conditions' OR (concurrent AND chronic AND medical AND conditions) OR 'multimorbidity' OR 'multimorbidity'/exp OR multimorbidity OR 'multiple chronic condition'/exp OR 'multiple chronic condition' OR (multiple AND chronic AND ('condition' OR 'condition'/exp OR condition)) OR 'multiple chronic diseases'/exp OR 'multiple chronic diseases' OR (multiple AND chronic AND ('diseases' OR 'diseases'/exp OR diseases)) OR 'multiple chronic disorders'/exp OR 'multiple chronic disorders' OR (multiple AND chronic AND ('disorders' OR 'disorders'/exp OR disorders)) OR 'multiple chronic health conditions'/exp OR 'multiple chronic health conditions' OR (multiple AND chronic AND ('health' OR 'health'/exp OR health) AND conditions) OR 'multiple chronic illnesses'/exp OR 'multiple chronic illnesses' OR (multiple AND chronic AND illnesses) OR 'multiple chronic medical conditions'/exp OR 'multiple chronic medical conditions' OR (multiple AND chronic AND medical AND conditions) OR 'simultaneous chronic illnesses'/exp OR 'simultaneous chronic illnesses' OR (simultaneous AND chronic AND illnesses) OR 'simultaneous chronic medical conditions'/exp OR 'simultaneous chronic medical conditions' OR (simultaneous AND chronic AND medical AND conditions)

#4

('epidemiology'/exp OR epidemiology OR 'clinical epidemiology'/exp OR 'clinical epidemiology' OR (('clinical'/exp OR clinical) AND ('epidemiology'/exp OR epidemiology)) OR 'confounding factors' OR (('confounding'/exp OR confounding) AND factors AND ('epidemiology'/exp OR epidemiology)) OR 'confounding factors, epidemiologic'/exp OR 'confounding factors, epidemiologic' OR (('confounding'/exp OR confounding) AND factors, AND epidemiologic) OR 'controlled before after studies'/exp OR 'controlled before after studies' OR (controlled AND before AND after AND ('studies'/exp OR studies)) OR (('controlled before' OR (controlled AND before)) AND ('after studies' OR (after AND ('studies'/exp OR studies)))) OR (('controlled before' OR (controlled AND before)) AND ('after study' OR (after AND ('study'/exp OR study)))) OR 'controlled before-after studies'/exp OR 'controlled before-after studies' OR (controlled AND 'before after' AND ('studies'/exp OR studies)) OR 'effect modifier, epidemiologic'/exp OR 'effect modifier, epidemiologic' OR (effect AND modifier, AND epidemiologic) OR (effect AND modifiers AND ('epidemiology'/exp OR epidemiology)) OR 'effect modifiers' OR (effect AND modifiers AND ('psychology'/exp OR psychology)) OR 'environmental epidemiology'/exp OR 'environmental epidemiology' OR (environmental AND ('epidemiology'/exp OR epidemiology)) OR 'epidemiologic confounding factors'/exp OR 'epidemiologic confounding factors' OR (epidemiologic AND ('confounding'/exp OR confounding) AND factors) OR 'epidemiologic effect modifier'/exp OR 'epidemiologic effect modifier' OR (epidemiologic AND effect AND ('modifier'/exp OR modifier)) OR 'epidemiologic factors'/exp OR 'epidemiologic factors' OR (epidemiologic AND factors) OR 'epidemiologic methods'/exp OR 'epidemiologic methods' OR (epidemiologic AND ('methods'/exp OR methods)) OR 'epidemiologic research'/exp OR 'epidemiologic research' OR (epidemiologic AND ('research'/exp OR research)) OR 'epidemiologic research design'/exp OR 'epidemiologic research design' OR (epidemiologic AND ('research'/exp OR research) AND ('design'/exp OR design)) OR 'epidemiologic studies'/exp OR 'epidemiologic studies' OR (epidemiologic AND ('studies'/exp OR studies)) OR 'epidemiologic study characteristics'/exp OR 'epidemiologic study characteristics' OR (epidemiologic AND ('study'/exp OR study) AND characteristics) OR 'epidemiologic study characteristics as topic'/exp OR 'epidemiologic study characteristics as topic' OR (epidemiologic AND ('study'/exp OR study) AND characteristics AND as AND topic) OR 'epidemiologic survey'/exp OR 'epidemiologic survey' OR (epidemiologic AND ('survey'/exp OR survey)) OR 'epidemiological research'/exp OR 'epidemiological research' OR (epidemiological AND ('research'/exp OR research)) OR 'epidemiometry'/exp OR epidemiometry OR 'historically controlled study'/exp OR 'historically controlled study' OR (historically AND controlled AND ('study'/exp OR study)) OR 'interrupted time series analysis'/exp OR 'interrupted time series analysis' OR (interrupted AND ('time'/exp OR time) AND series AND ('analysis'/exp OR analysis)) OR 'precipitating factors'/exp OR 'precipitating factors' OR (precipitating AND factors)) AND ('sampling studies'/exp OR 'sampling studies' OR (('sampling'/exp OR sampling) AND ('studies'/exp OR studies)))

#5

#1 AND (#2 OR #3 OR #4)

#6

#5 NOT review
